# Artificial intelligence-assisted fast screening cervical high grade squamous intraepithelial lesion and squamous cell carcinoma diagnosis and treatment planning

**DOI:** 10.1038/s41598-021-95545-y

**Published:** 2021-08-10

**Authors:** Ching-Wei Wang, Yi-An Liou, Yi-Jia Lin, Cheng-Chang Chang, Pei-Hsuan Chu, Yu-Ching Lee, Chih-Hung Wang, Tai-Kuang Chao

**Affiliations:** 1 Graduate Institute of Biomedical Engineering, National Taiwan University of Science and Technology, Taipei, Taiwan; 2 Graduate Institute of Applied Science and Technology, National Taiwan University of Science and Technology, Taipei, Taiwan; 3 Department of Pathology, Tri-Service General Hospital, Taipei, Taiwan; 4 Institute of Pathology and Parasitology, National Defense Medical Center, Taipei, Taiwan; 5 Department of Gynecology and Obstetrics, Tri-Service General Hospital, Taipei, Taiwan; 6 Graduate Institute of Medical Sciences, National Defense Medical Center, Taipei, Taiwan; 7 Department of Otolaryngology-Head and Neck Surgery, Tri-Service General Hospital, National Defense Medical Center, Taipei, Taiwan

**Keywords:** Cancer screening, High-throughput screening, Machine learning, Biomedical engineering, Cervical cancer

## Abstract

Every year cervical cancer affects more than 300,000 people, and on average one woman is diagnosed with cervical cancer every minute. Early diagnosis and classification of cervical lesions greatly boosts up the chance of successful treatments of patients, and automated diagnosis and classification of cervical lesions from Papanicolaou (Pap) smear images have become highly demanded. To the authors’ best knowledge, this is the first study of fully automated cervical lesions analysis on whole slide images (WSIs) of conventional Pap smear samples. The presented deep learning-based cervical lesions diagnosis system is demonstrated to be able to detect high grade squamous intraepithelial lesions (HSILs) or higher (squamous cell carcinoma; SQCC), which usually immediately indicate patients must be referred to colposcopy, but also to rapidly process WSIs in seconds for practical clinical usage. We evaluate this framework at scale on a dataset of 143 whole slide images, and the proposed method achieves a high precision 0.93, recall 0.90, F-measure 0.88, and Jaccard index 0.84, showing that the proposed system is capable of segmenting HSILs or higher (SQCC) with high precision and reaches sensitivity comparable to the referenced standard produced by pathologists. Based on Fisher’s Least Significant Difference (LSD) test (P < 0.0001), the proposed method performs significantly better than the two state-of-the-art benchmark methods (U-Net and SegNet) in precision, F-Measure, Jaccard index. For the run time analysis, the proposed method takes only 210 seconds to process a WSI and is 20 times faster than U-Net and 19 times faster than SegNet, respectively. In summary, the proposed method is demonstrated to be able to both detect HSILs or higher (SQCC), which indicate patients for further treatments, including colposcopy and surgery to remove the lesion, and rapidly processing WSIs in seconds for practical clinical usages.

## Introduction

According to projections by the World Health Organization (WHO), cervical cancer affects more than 300,000 people per year, with more than 85% of such deaths occurring in less developed countries in recent decades. Each minute, one woman is diagnosed with cervical cancer, namely one of the most common cancers in women’s health today^1^. Dysplasia could be detected earlier before cervical cancer grows. The sooner this is detected, the easier to cure cervical cancer. Cervical cancer is completely preventable and curable if pre-cancer signs are identified and treated at an early stage^2^. Pap smear is commonly used for medical diagnosis of the cervix to monitor cervical cancer and other diseases. The method of Pap smear is to obtain a small number of cervical cell samples, make a cell smear, observe the cells under the microscope for abnormalities, and then diagnose cervical disease. The sample usually screened by cytotechnologists to examine the cell sample for signs of malignancies. Through this procedure, medical experts could both find proof of invasive cancer and detect certain cancer precursors, allowing for early and effective treatment. According to the WHO classification for cervical squamous lesion, the initial and mild stage of precancer is termed as mild dysplasia, which later advances to the next stage called moderate dysplasia, followed by severe dysplasia and squamous cell carcinoma in situ, and finally to invasive squamous cell carcinoma (SQCC) that invades other parts of the body.

In Cervical Intraepithelial Neoplasia (CIN) system, mild dysplasia is classified as CIN1, moderate dysplasia as CIN2, severe dysplasia, and squamous cell carcinoma in situ as CIN3, and the final stage as invasive SQCC. The Bethesda system is a standard system worldwide in cytological reporting of the cases in diagnosing cervical lesions. This system further eliminates the subclassification of CIN by categorizing CIN1 as low-grade squamous intraepithelial lesion (LSIL) and CIN2 and CIN3 as high-grade squamous intraepithelial lesion (HSIL) and the last stage as invasive cancer^3^. Atypical squamous cells (ASC) divides into two subcategories: atypical squamous cells of undetermined significance (ASC-US) and atypical squamous cells, which cannot exclude a high-grade squamous intraepithelial lesion (ASC-H). ASC-H is the less common qualifier, accounting for 5 to 10% of all ASC cases, but the risk of the potentially high-grade lesion is higher in this category than in ASC-US. This diagnostic category includes a mixture of real HSILs and its mimics^4^. Women with HSIL, for whom the risk of cancer is high, are immediately referred for colposcopy and, if the lesion is confirmed, surgery is required to remove the tumor^5^. The rate of concurrent and subsequent HSILs on follow up of ASC-H is reported as 29–75%, and it is recommended that women diagnosed with ASC-H also should be referred for colposcopy^4^. The detection of LSIL or ASC-US may lead to a follow-up smear being taken after a shorter time interval than the normal 2–3 years. Pap smears can greatly reduce the incidence of cervical cancer. During the Pap smear examination, cytopathologists manually scan and inspect the whole slide at the microscope using magnification at 10$\times$, and when something suspicious is seen, detailed inspection is conducted at magnification 40$\times$. This process typically involves checking thousands of cells^6^. Manual analysis of the Pap smear images requires a large amount of well trained manpower, which is extremely expensive, time-consuming, laborious, and error-prone and not available in many hospitals. More importantly, if malignant cells are carelessly neglected during the manual screening process, this will jeopardize the subsequent treatment plan, causing the patient miss the opportunity of early treatment and even more serious consequences. In addition, high inter-observer variability substantially affects productivity in routine pathology and is especially ubiquitous in diagnostician-deficient medical centers^7^. With an increase in computing power and advance in imaging technologies, deep learning is being implemented for the diagnosis and classification of cervical lesions. Deep learning has been used for the detection of diseases, such as skin cancer^8^, lung cancer^9^, cardiac arrhythmia^10^, retinal disease^11^, intracranial hemorrhage^12^, neurological problems^13^, autism^14^, kidney disease^15^ and psychiatric problems^16^.

In 2019, Araújo et al.^17^ applied convolution neural networks (CNNs) to segment LSILs or ASCUSs using small size cervical cell images (1392 $\times$ 1040 pixels) acquired by manually identified regions of interests from microscopy, and Lin et al.^18^ applied CNNs to classify abnormal cells using single cervical cell image with averaged size (110 $\times$ 110 pixels), which is carefully prepared by manual localization and extraction of microscopic images. However, detection of LSILs or ASCUSs may only lead to a more shorter follow up interval, and both methods^17,18^ require manual intervention to locate and acquire single-cell images or images of regions of interests, and thus the two methods could not be utilized for fully automatic WSI analysis in cervical HSILs or higher (SQCC) examination. To the authors’ best knowledge, there has been no published work on automated cervical HSILs or higher (SQCC) analysis using whole slide images (WSIs) of conventional Pap smear samples for practical usages.

**Figure 1:**
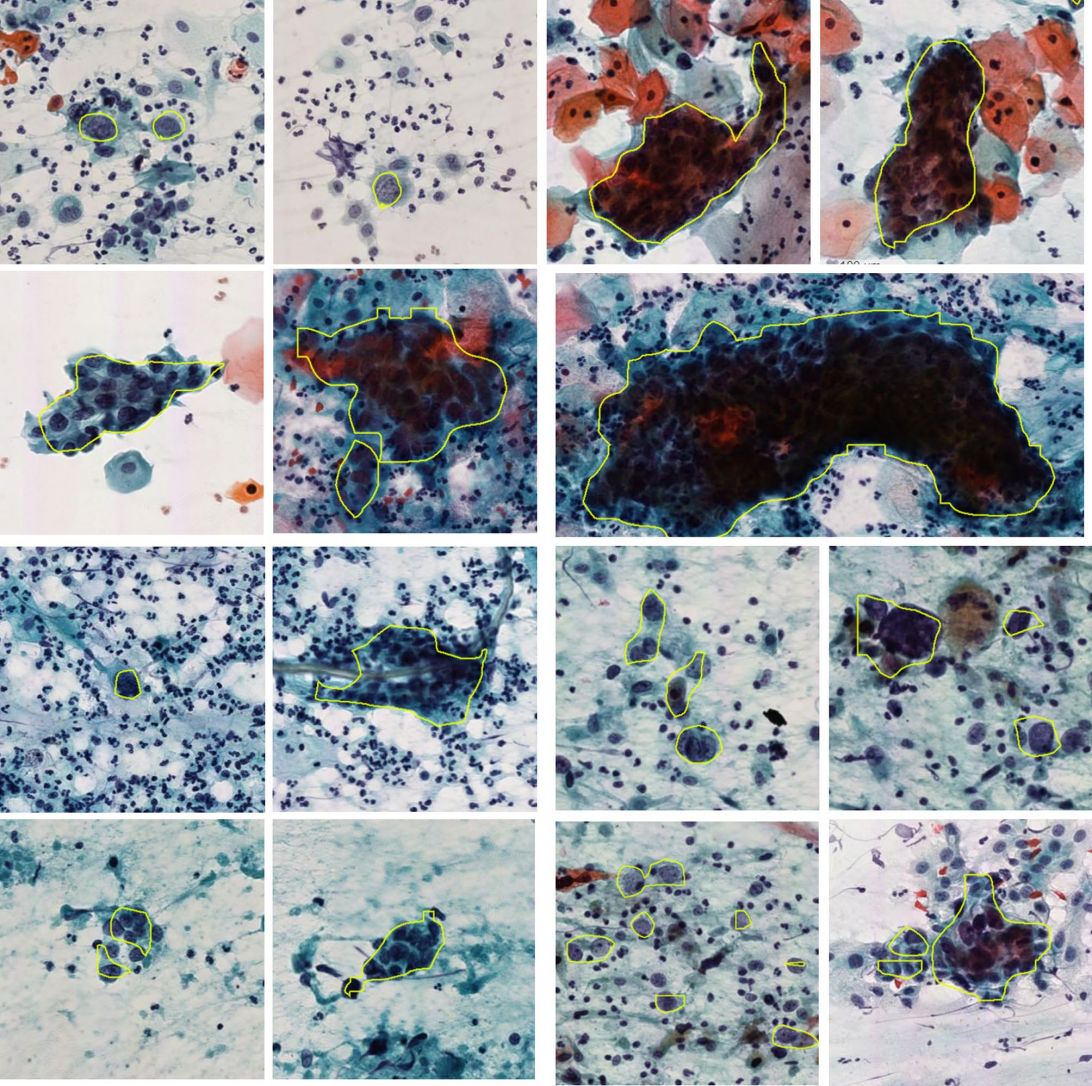
Segmentation results of randomly selected examples by the proposed method where the HSILs are highlighted in yellow.

In this study, we propose a deep learning based cervical HSILs or higher (SQCC) diagnosis and treatment planning system using Papanicolaou staining, enabling automatic examination of cervical smear on WSIs and identification and quantification of HSILs or higher (SQCC) for further treatment suggestion. If HSILs or higher (SQCC) are detected in a patient Pap smear sample, the further clinical step is to perform the biopsy, large loop excision of the transformation zone (LEEP/LLETZ), and cold knife conization of the cervix to achieve both diagnosis and treatment. The collection of WSIs stained by Papanicolaou was obtained from Tri-service general hospital, Taipei, Taiwan, and a reference standard is produced by manual annotations of HSILs or higher (SQCC) by pathologists. In evaluation, as this is the first study on automatic HSILs or higher (SQCC) segmentation, we compare the proposed method with Araújo et al.’s approach^17^ for LSIL segmentation as well as two state-of-the-art deep learning methods (U-net^19^ and SegNet^20^) as shown in Fig. 1.

## Data and results

### Material

**Figure 2:**
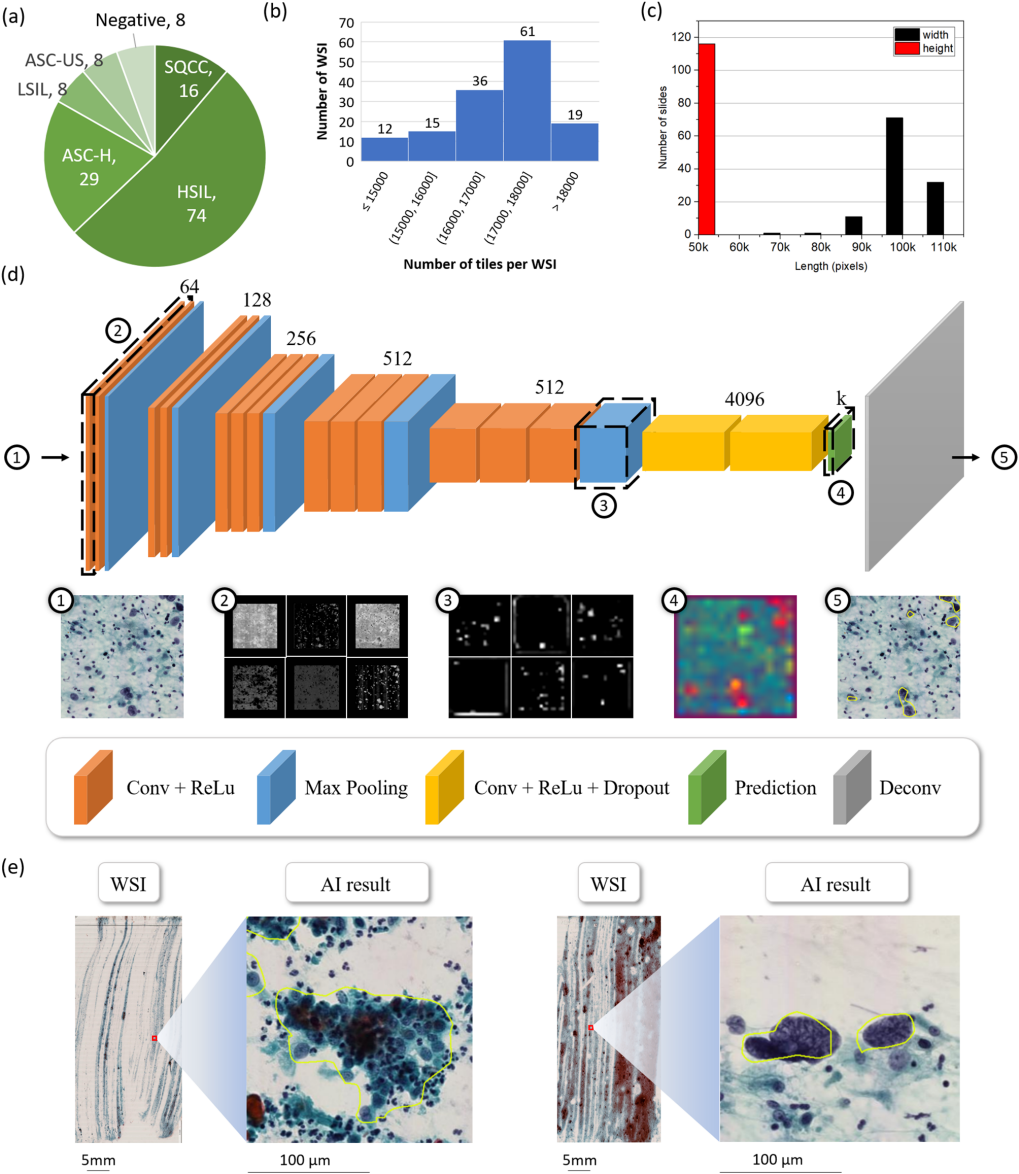
Data information and illustration of the proposed network framework. (**a**) The number of WSIs per category. (**b**) Distribution of the tile numbers per WSI. (**c**) Size distribution of the WSIs with width and height as black and red. (**d**) Illustration of the proposed modified FCN structure, and the feature maps of the sampling layers. (**e**) The results of the proposed method in WSIs.

De-identified, digitized whole-slide images of conventional Pap smear samples were obtained from the tissue bank of the Department of Pathology, Tri-Service General Hospital, National Defense Medical Center, Taipei, Taiwan (n = 143 patients). A research ethics approval has been gained from the research ethics committee of the Tri-Service General Hospital (TSGHIRB No.1-107-05-171 and No.B202005070), and informed consent is formally waived by the approving committee. The data were de-identified and used for a retrospective study without impacting patient care. All methods were carried out in accordance with relevant guidelines and regulations. Cervical scrapings were collected for cytological diagnosis by gynecologists. The slides were prepared and stained by the Pap method according to the usual laboratory protocol. The screening of cytology slides was first performed by the pool of cytotechnologists, and a pathologist always confirmed abnormal results. Cytology was performed using TBS 2014. A series of negative (n = 8), ASC-US (n = 8), LSIL (n = 8), ASC-H (n = 29), HSIL (n = 74), or higher (SQCC, n = 16), and the number of per category in the dataset as shown in Fig. 2a. All patients were treated and followed by the standard clinical protocol. The patients with ASC-US underwent a repeat Pap smear within 1 year, while the patients with ASC-H, HSIL or SQCC underwent colposcopy-directed cervical biopsy and subsequent therapy when indicated. WSIs are digitized glass slides from scanning devices. All stained slides were scanned using Leica AT Turbo (Leica, Germany), at 20$\times$ objective magnification. The network was instead trained and tested using non-overlapping tiles (512–512 pixels) obtained from the WSIs. Distribution of the tile numbers per WSIs as shown in Fig. 2b. In computational pathology, the massive size of WSIs is one of the challenges. For automatic analysis the average size of WSIs is 91,257 $\times$ 41,546 pixels, 45.93 $\times$ 20.91 mm in our dataset, and the size distribution of the WSIs as shown in Fig. 2c. The proposed network structure with the associated outputs of the selected layers are shown in Fig. 2d. A WSI generally contains billions of pixels, while the regions of interest could be as small as a few thousands of pixels (see Fig. 2e). We collected slide-level reviews and region-level annotations from pathologists. Slide-level reviews categorize each slide into a group of higher than LSIL including ASC-H, HSIL and SQCC. Region-level annotations represent specific HSILs or higher (SQCC) within a slide. The information for the dataset is shown in Table 1.

**Table 1:** Data distribution and ratio of sampling tissue for AI training.

Category	Types	Number of patients’ WSIs		Ratio of sampling tissue in training	
		Training set	Testing set	With respect to training set	With respect to whole set
$\ge$ ASC-H	SQCC	15	1	0.00011	0.00010
	HSIL	57	17	0.00006	0.00005
	ASC-H	25	4	0.00004	0.00003
< ASC-H	LSIL	0	8	0	0
	ASC-US	0	8	0	0
	Negative	0	8	0	0
Total (%)		97 (68%)	46 (32%)	0.00006	0.00004

### Experimental set-up and implementation details

In evaluation, the whole slide images were randomly split into two sets: 68% for training and 32% for testing. As shown in Table 1, the training set consists of 25 ASC-H, 57 HSIL, and 15 SQCC cases, and the tiles annotated and sampled for building AI models account for 0.006% of the training WSIs and for 0.004% of the whole data set, respectively. Moreover, the proposed framework is initialized using VGG16 model, and stochastic gradient descent (SGD) optimization and the cross entropy loss function are utilized. In addition, the network training parameters of the proposed method, including the learning rate, dropout ratio, and weight decay, are set to $1\times 10^{-10}$, 0.5, and 0.0005, respectively. The benchmark methods (U-net^19^ and SegNet^20^) are implemented using the keras impelementation of images segmentation models by Gupta et al.^21^. For training, the benchmark methods (U-net^19^ and SegNet^20^) are initialized using a pre-trained VGG16 model with the networks optimized using Adadelta optimization, and the cross entropy function is used as a loss function. In addition, the network training parameters of U-Net and SegNet, including the learning rate, dropout ratio, and weight decay, are set to 0.0001, 0.2, and 0.0002, respectively. The testing set accounting for 32% of the whole set, consisted of 8 Negative, 8 ASC-US, 8 LSIL, 4 ASC-H, 17 HSIL, and 1 SQCC. Further details on the data set could be found in Table 1. To assess the performance of the proposed method, we compared the AI segmentation results with the reference standard annotated by pathologists. Further quantitative evaluation details are described in the next section.

### Evaluation method

In this study, two state-of-the-art deep learning methods (U-net^19^ and SegNet^20^) and Araújo et al.’s method^17^ are adopted as the benchmark approaches, and we compare the computing speed, precision, recall, F-measure, Jaccard index, which is often used in semantic segmentation to validate the pixel-level labeling performance^22–26^, of the proposed method and the benchmark approaches. Objects are classified into one of the four categories: TP, true positive; TN, true negative; FP, false positive; FN, false negative. The evaluation metrics are formulated as follows.

1 $$\begin{aligned} Precision= & {} \frac{TP}{TP~+~FP} \end{aligned}$$

2 $$\begin{aligned} Recall= & {} \frac{TP}{TP~+~FN}\end{aligned}$$

3 $$\begin{aligned} F\text {-}measure= & {} 2~\times ~\frac{Precision~\times ~Recall}{Precision~+~Recall}~=~\frac{2TP}{2TP~+~FP~+~FN}\end{aligned}$$

4 $$\begin{aligned} Jaccard~Index= & {} \frac{F\text {-}measure}{~\left( ~2~-~F\text {-}measure~ \right) ~}~=~\frac{TP}{TP~+~FP~+~FN} \end{aligned}$$

### Quantitative evaluation and statistical analysis

**Figure 3:**
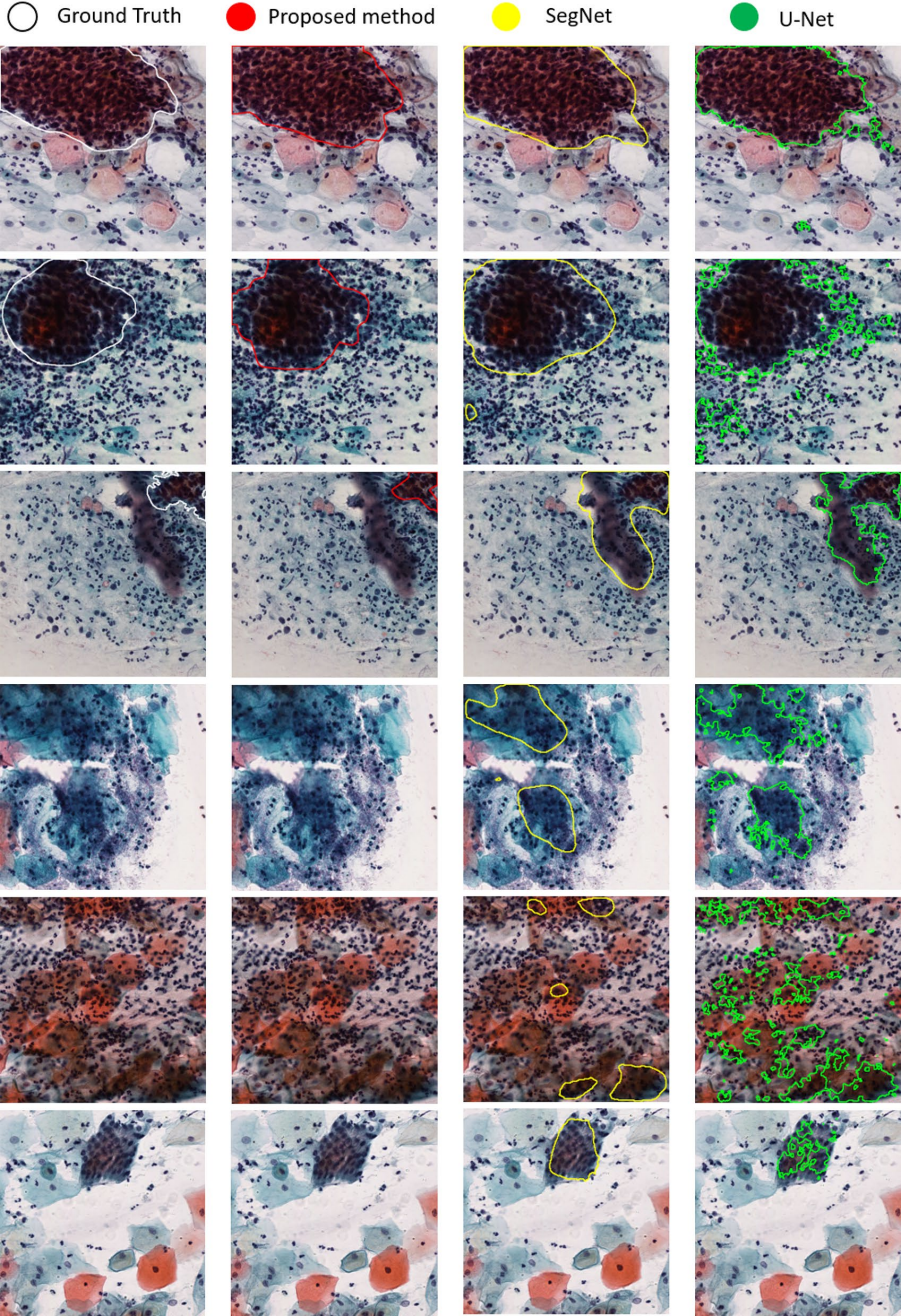
Qualitative segmentation results by the proposed method and two benchmark approaches for HSIL detection.

In evaluation, as this is the first study on automatic HSILs or higher (SQCC) segmentation, we compare the proposed method with Araújo et al.’s approach^17^ for LSIL segmentation on small image patches as well as two state-of-the-art deep learning methods (U-net^19^ and SegNet^20^) as shown in Table. 2 where the reported numbers of Araújo et al.^17^ are referred. The results show that the proposed method achieves high precision 0.93, recall 0.90, F-measure 0.88 and Jaccard index 0.84 and outperforms the three benchmark approaches in all four measurements. In comparison, the U-Net model obtains precision 0.15, recall 0.70, F-measure 0.17 and Jaccard 0.12; SegNet model obtains precision 0.26, recall 0.88, F-measure 0.27 and Jaccard 0.23; Araújo et al.’s approach^17^ is with precision 0.66, recall 0.72, F-measure 0.69 and Jaccard 0.53. Furthermore, Fig. 3 presents qualitative segmentation results by the proposed method and two benchmark approaches for HSILs or higher (SQCC) detection. The results demonstrate the high precision, efficiency and reliability of the proposed model.

**Table 2:** Quantitative comparison with benchmark methods.

	Target	Data type	Aver. size (pixel)	Precision	Recall	F-measure	Jaccard
Proposed method	HSIL	WSIs	91257 41546	**0.93**	**0.90**	**0.88**	**0.84**
U-net^19^	HSIL	WSIs	91257 41546	0.15	0.70	0.17	0.12
SegNet^20^	HSIL	WSIs	91257 41546	0.26	0.88	0.27	0.23
Araújo et al.^17^	LSIL/ASCUS	Image patches	1383 1036	0.66	0.72	0.69	0.53

The proposed method is significantly better than the benchmark approaches (p<0.0001).

**Figure 4:**
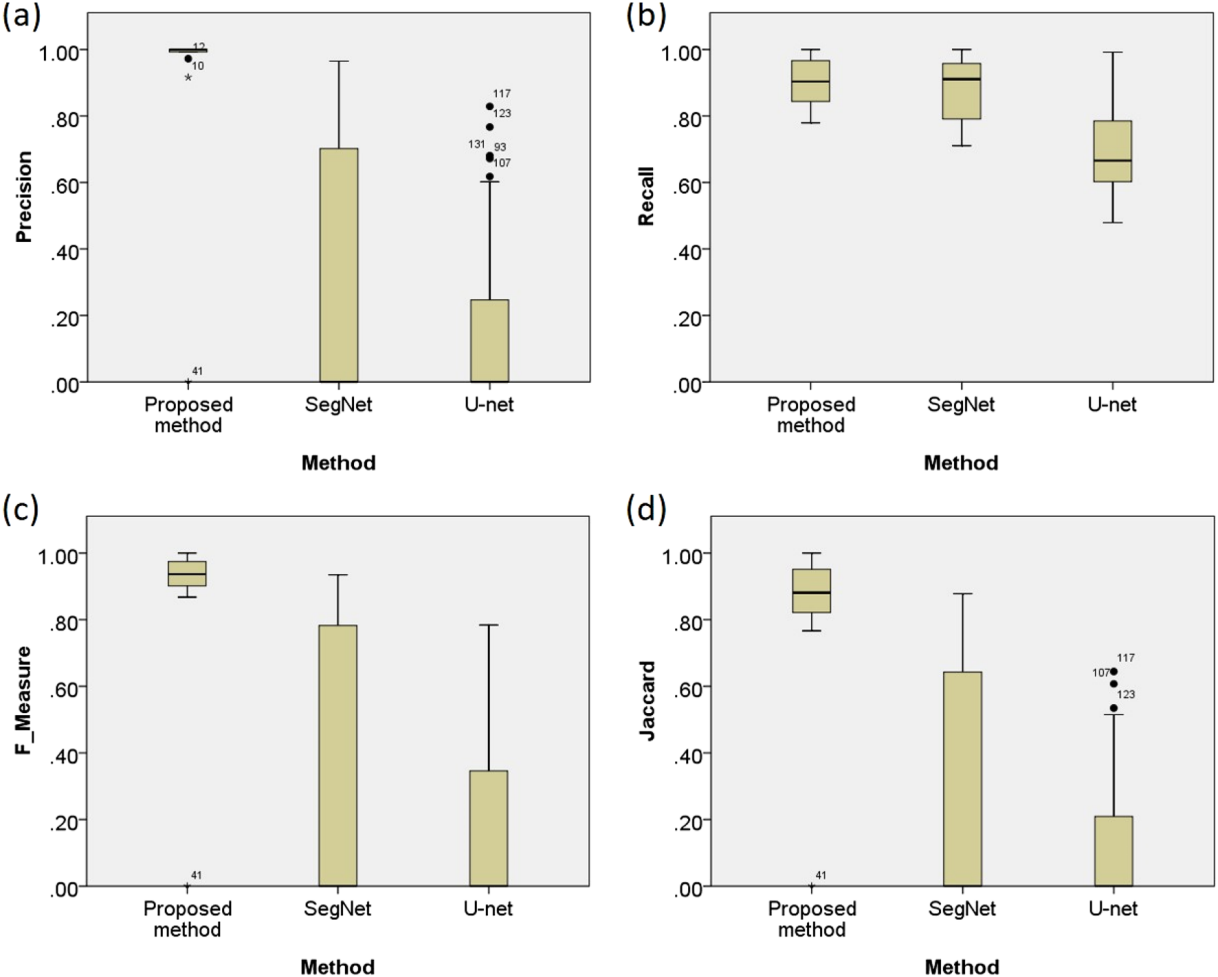
The box plot of quantitative evaluation results in HSILs or higher (SQCC) segmentation. The presented method works constantly well overall and outperforms the state-of-the-art benchmark methods. Outliers greater than $1.5\times$ and $3\times$ the interquartile range are marked with a dot and a asterisk, respectively.

Table 3 presents detailed quantitative evaluation results in HSILs or higher (SQCC) segmentation for all samples and for separate evaluation on samples with high-grade lesions and samples with low-grade lesions or negative. The box plots of the quantitative evaluation results for all samples are provided in Fig. 4, showing that the proposed method consistently performs well with high precision, recall, F-measure. Jaccard and specificity and outperforms the two state of the art deep learning methods, i.e. SegNet and U-net. The experimental results show that the two benchmark methods (U-net^19^ and SegNet^20^) perform poor in detecting HSILs or higher (SQCC), obtaining precision, recall, F-Measure, Jaccard index were $<26\%,<88\%,<27\%, <23\%$ on average, respectively.

Furthermore, for statistical analysis, using SPSS software^27^, the quantitative scores were analyzed with the Fisher’s Least Significant Difference (LSD) to compare multiple methods (see Table 4). In precision, the presented method achieves 92.94% averaged and significantly outperforms both benchmark methods based on LSD tests ($P < 0.0001$). In recall, the presented method achieves 89.85% averaged and significantly outperforms the U-net method based on LSD tests ($P < 0.0001$). In F-Measure, the presented method achieves 88.21% averaged and significantly outperforms both benchmark methods based on LSD tests ($P < 0.0001$). In Jaccard index, the presented method achieves 83.57% averaged and significantly outperforms both benchmark methods based on LSD tests ($P < 0.0001$). Figure 1 presents more segmentation results of randomly selected examples of by the proposed method.

**Table 3:** Quantitative evaluation of the proposed method and two benchmark methods (U-Net and SegNet) in segmenting of HSILs or higher (SQCC).

	Proposed method			SegNet^20^			U-net^19^		
	All	$\ge$ HSIL	$\le$ LSIL	All	$\ge$ HSIL	$\le$ LSIL	All	$\ge$ HSIL	$\le$ LSIL
Precision	**0.9294**	**0.9913**	0^a^	0.2573	0.5145	0^a^	0.1528	0.3195	0^a^
Recall (sensitivity)	**0.8987**	**0.8987**	NaN	0.8760	0.8760	NaN	0.7053	0.7053	NaN
F-measure	**0.8831**	**0.9420**	0^a^	0.2670	0.5341	0^a^	0.1691	0.3536	0^a^
Jaccard	**0.8356**	**0.8913**	0^a^	0.2259	0.4518	0^a^	0.1220	0.2550	0^a^
Specificity	**1.0000**	**1.0000**	**1.0000**	0.9833	0.9709	0.9946	0.5752	0.7327	0.4308

The proposed method is significantly better than the benchmark approaches (p<0.0001).

^a^As there are no positive in these samples, the value is computed as 0.

**Table 4:** Multiple comparisons for the segmentation of HSILs or higher (SQCC): Fisher’s LSD test.

LSD multiple comparisons							
Dependent variable	(I) Method	(J) Method	Mean difference (I-J)	Std. error	Sig.	95% CI	
						Lower bound	Upper bound
Precision	Proposed method	U-net^19^	0.77696^a^	0.09093	< 0.0001	0.5966	0.9573
		SegNet^20^	0.67273^a^	0.09146	< 0.0001	0.4913	0.8541
Recall	Proposed method	U-net^19^	0.19469^a^	0.04242	< 0.0001	0.1091	0.2803
		SegNet^20^	0.02283	0.04242	0.593	− 0.0628	0.1084
F-measure	Proposed method	U-net^19^	0.71301^a^	0.09314	< 0.0001	0.5283	0.8977
		SegNet^20^	0.61492^a^	0.09369	< 0.0001	0.4291	0.8007
Jaccard	Proposed method	U-net^19^	0.71348^a^	0.07790	< 0.0001	0.5590	0.8680
		SegNet^20^	0.61008^a^	0.07836	< 0.0001	0.4547	0.7655

^a^The proposed method is significantly better than the benchmark methods (U-net^19^ and SegNet^20^) ($P < 0.0001$).

### Run time analysis

Due to the enormous size of WSIs, the computational time for WSI analysis is critical for practical clinical usage. Therefore, we analyzed the AI inference time using different hardware settings (see Table 5a). For the run time analysis, the proposed method takes only 210 seconds to process a WSI and is 20 times faster than U-Net and 19 times faster than SegNet, respectively (see Table 5b). In comparison with Araújo et al.’s method^17^, the proposed method processes 923,520 more pixels per second, and Araújo et al.’s method requires twice of hardware memory (251 GB) than the proposed method (128 GB). Overall, the proposed method is demonstrated to be able to both detect HSILs, which indicate patients for further treatments, including colposcopy and surgery to remove the lesion, and rapidly processing WSIs in seconds for practical clinical usages.

**Table 5:** Comparison on (a) hardware and (b) computing efficiency.

Method	CPU	RAM (GB)	GPU
**(a)**			
Proposed method	Intel Xeon Gold 6134 CPU @ 3.20GHz 16	128	4 GeForce GTX 1080Ti
U-net^19^	Intel Xeon CPU E5-2650 v2 @ 2.60GHz 16	32	1 GeForce GTX 1080Ti
SegNet^20^	Intel Xeon CPU E5-2650 v2 @ 2.60GHz 16	32	1 GeForce GTX 1080Ti
Araúj et al.^17^	Intel Xeon E5-2643 @ 3.40 GHz 6	251	4 GeForce GTX Titan-X

Method	Inference time in seconds (per WSI^a^)	Inference pixels (per second)	
**(b)**			
Proposed method	210	21,604,662	
U-net^19^	4370	1,038,196	
SegNet^20^	4024	1,127,500	
Araúj et al.^17^	219$^{l}$	20,681,142$^{l}$	

^a^The size of the WSI in this evaluation is 4,536,979,200 pixels (99,600$\times$45,552 pixels). ^b^Araúj et al.’s method^17^ takes 0.07 s for processing a patch with 1392 $\times$ 1040 pixels; ^b^219 s = $\left\lfloor \frac{99,600 \times 45,552}{1,392 \times 1040} \times 0.07 \right\rfloor$; 20,681,142 pixels = $\left\lfloor 1392 \times 1040 \div 0.07 \right\rfloor$.

## Discussion

To the authors’ best knowledge, this is the first work on automated cervical HSILs or higher (SQCC) analysis of WSIs on conventional Pap smear samples for practical usages. Our study demonstrates that the proposed new cervical Pap smear diagnosis system could be used to assist in automatic detection and quantification of cervical HSILs or higher (SQCC) from WSIs. The proposed method achieved a high precision 0.93, recall 0.90, F-measure 0.88, and Jaccard index 0.84, and capable of unambiguously segmenting HSILs or higher (SQCC), showing that the proposed system is robust and capable of segmenting HSILs or higher (SQCC) with high precision and reaches sensitivity comparable to the referenced standard produced by pathologists. Moreover, the proposed method significantly outperforms two state of the art deep learning approaches ($$P<0.001$$). Cervical cancer develops through persistent infection with high-risk human papilloma virus (HR-HPV) and is a leading cause of death among women worldwide^28^. Regular screening strategies using HR-HPV, Pap smear and colposcopy alone or in combination can prevent the onset and development of cervical cancer^28^. Cervical cancer incidence can be reduced by as much as 90% where screening quality and coverage are high^29^. In 2018, the United States Preventive Services Task Force (USPSTF) updated its screening guidelines. In addition to continuing to recommend triennial cytology (Pap test) for women between 21 and 29 years old, then continue with triennial cytology or increase HR-HPV testing every 5 years between 30 and 65 years old^30^. The major contribution of our proposed method in a cervical Pap smear screening workflow compared to manual cytology reading is that it reduces on the time required by the cytotechnician to screen many pap-smears by eliminating the obvious normal ones, hence more time can be put on the suspicious slides. In recent decades, although the conventional Pap smear method has been the mainstay of the screening procedures. However, this technique is not without limitations, because the sensitivity and specificity are relatively low. Liquid-based cytology (LBC) was introduced in the 1990s and was initially considered a better tool for processing cervical lesions. But now it has been found that LBC is more superior to conventional smears only with respect to a lesser number of unsatisfactory smears. There is no significant difference in the detection of epithelial cell abnormalities between the two methods^31^. LBC is being widely used in the United States, European countries, and many other developed nations. Although these approaches appear better clarity, uniform spread of smears, less time for screening and better handling of hemorrhagic and inflammatory samples^32^, but they are expensive and rely heavily on technology^33^. To consider the cost effectiveness and health insurance policy , the conventional Pap method is more feasible in our country. Although HPV testing is more sensitive to detect cervical precancerous lesions and cancers earlier than cytology, there are currently costs, infrastructure considerations and specificity issues that limit its use in low- and middle-income countries^34^. The high frequency of transient HPV infection among women younger than 30 years can lead to unnecessary follow-up diagnostic and treatment interventions with potential for harm^35^. For the HR-HPV screening, the Food and Drug Administration (FDA) approved cobas HPV testing. This test detects HPV types 16, 18, and 26 and additional HR-HPV types^36^. Despite the reported high sensitivity (86%) and negative predictive value (82%) of HR-HPV testing^37^, some HSIL can still be missed^38–40^. The low specificity (31%) and positive predictive value (37%) even make the situation worse because they lead to more patients undergoing unnecessary referrals^37^. Recent studies have shown the correlation between epigenetics and development and progression of cervical cancer^41^. Increased methylation of host genes has been observed in women with cervical precancer and cancer. Several of these genes have been evaluated as candidates for triage of HPV-positive women. However, more longitudinal studies are needed to prove the longitudinal safety of negative methylation result^42^. Vaccination against HPV is a possible long-term solution for eradicating cervical cancer in developing countries, where a prophylactic HPV vaccine has already been approved. However, knowledge and awareness about cervical cancer, HPV, and the efficacy of the HPV vaccine in the prevention of cervical cancer are very low in the world. The low level of knowledge about HPV is considered to be the major hurdle for the implementation HPV vaccination programs^30^. Automatic screening of Papanicolaou system has been available for more than 25 years, such as AutoPap 300^43^ and the PapNet^44^, which were approved by the United States FDA in 1998. Cytyc was approved by the FDA in 2007 with the ThinPrep imaging system^45^. Recent reviews indicate that the previous image analysis and machine learning techniques used for automatic Pap smear screening are flawed, resulting in low accuracy. The development of fully automatic screening technology that does not rely on the human judgment has yet to be fully realized^46^. A fast automated deep learning system enables high throughput analysis across a wide cohort of patients, and also helps to obtain a large amount of data to analyze the enormous dimensions of large gigapixel data of WSIs. There is limited research on automated analysis of cervical lesions on conventional Pap smear WSIs. Araújo et al.^17^ applied CNNs to segment LSIL or ASCUS using small size cervical cell images (1392 $$\times $$ 1040 pixels) acquired by manually identified regions of interests from microscopy, and Lin et al.^18^ applied CNNs to classify abnormal cells using single cervical cell image with average size (110 $$\times $$ 110 pixels), which is carefully prepared by manual localization and extraction of microscopic images. Both methods require manual intervention to locate and acquire single-cell images or images of regions of interest. In comparison, we developed a fast and fully automatic deep learning fast screening system, which is capable of detection and quantification of HSILs or higher (SQCC) on WSIs in seconds for cervical lesion diagnosis and treatment suggestion. Our data demonstrated that AI-assisted cytology could distinguish most of CIN2+ (higher than CIN2) cytology based on a high precision 0.93, recall 0.90, F-measure 0.88, and Jaccard index 0.84. Compare to the manual cytology reading, it is close to an effective use in clinical practice due to complete CIN2+ cells labeling in a short time, which aid cytologists or cytotechnologists in screening and labeling cervical high grade dysplastic cells more easily and quickly. There are still some weaknesses in AI based Pap smear screening. When atypical cervical cells are gathered in different planes, traditional microscopes can overcome the focus problem by turning the adjustment wheel, but AI is not easy to correctly classify, such as HSIL present in three-dimensional groups closely mimic shed endometrial cells or HSIL pattern resembling reparative change. Specimen with rare, small, high nuclear to cytoplasmic ratio HSIL cells may be problematic with AI regard to identifying the single HSIL cells. The Pap smear image sometimes contain overlapping hyperchromatic crowded groups which can interfere the AI cytological diagnosis. Although our proposed method can correctly find out CIN2+ cells, but it still needs cytologists to confirm this diagnosis and divided CIN2+ cells into moderate dysplasia, severe dysplasia, squamous cell carcinoma in situ, nonkeratinizing SQCC or keratinizing SQCC. Furthermore, the proposed system is demonstrated to be superior than two state-of-the art deep learning methods, i.e. U-Net^19^ and SegNet^20^, in precision, recall, F-measure, Jaccard index and computing efficiency based on the experimental results using LSD test ($$P < 
0.001$$). The precision, recall, F-measure and Jaccard index was calculated from a hospital-based, retrospective study using a research platform that may not be directly applicable to the clinical setting or to wider populations. The application of artificial intelligence may provide a new screening method of cervical Pap smear and warrants further validation in a larger population-based study in future work.

Our results show that the proposed AI assisted method with high sensitivity (0.9) and specificity (1.0) outperforms conventional Pap smear examination and HPV testing, overcoming the limitation of low sensitivity in conventional Pap smear slides using light microscopic examination and low specificity in HPV testing. Artificial intelligence assisted rapid screening has great potential to provide much faster and cheaper service in the future. The processing time, material and labor cost could be greatly reduced using artificial intelligence assisted rapid screening. The proposed fast screening deep learning based system could not only avoid misdiagnosis by human negligence but also resolve lengthy screening process. The proposed system is applicable for practical clinical usage worldwide for comprehensive screening and ultimately has an impact on the areas with high incidences of cervical cancer.

## Methods

**Figure 5:**
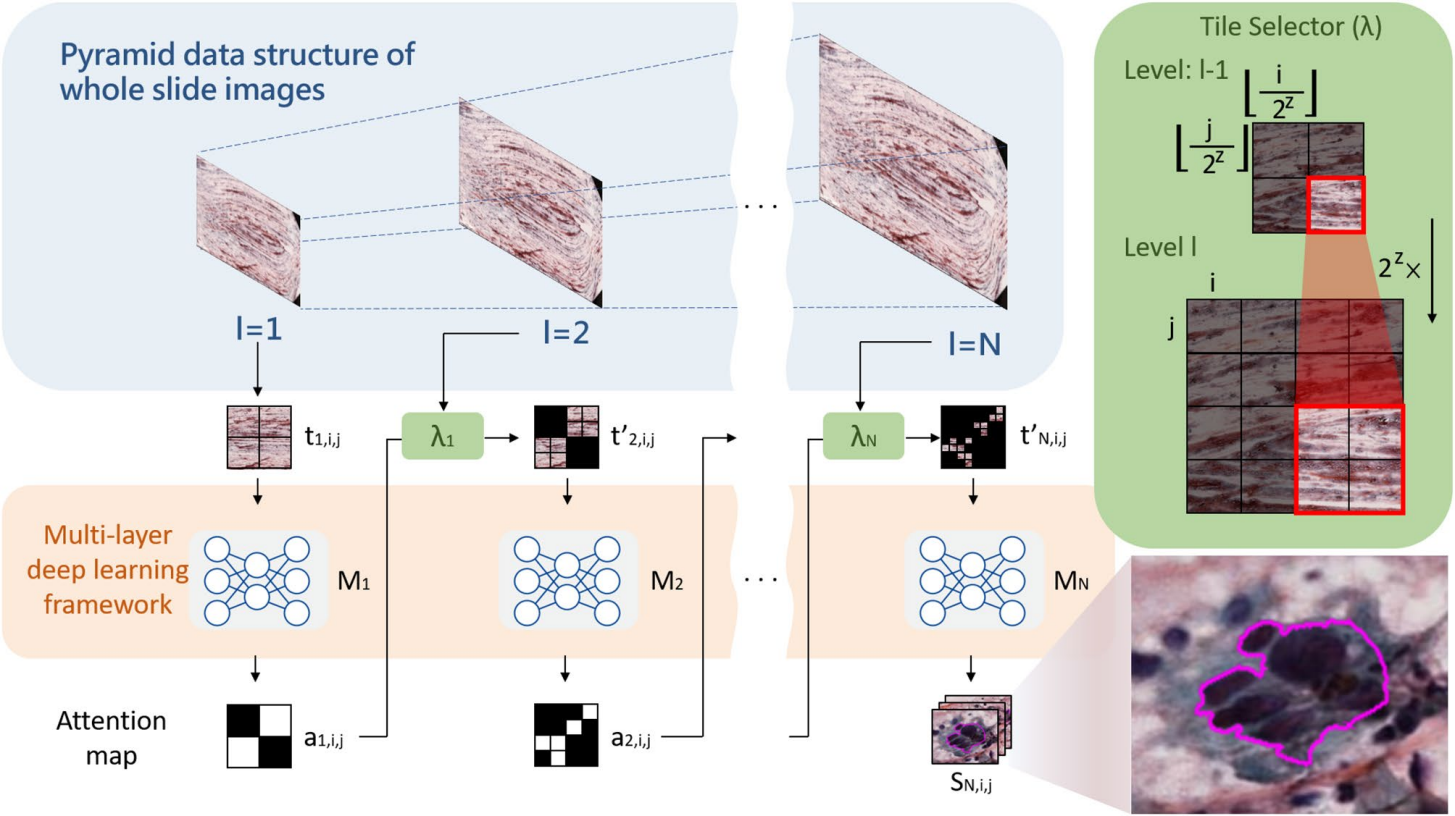
System framework. Each WSI is formatted into a tile-based pyramid data structure and the probability of HSILs at every level is generated by multi-layer deep learning framework. A multi-layer attention map is computed to select tiles of interest at every next level by a tile selector model $\lambda$, and the segmentation result of HSILs is generated by the last layer deep learning model $M_{N}(t'_{N,i,j}(x,y))$.

In this paper, we developed an efficient system to identify HSILs on WSIs with a cascaded multi-layer deep learning framework to improve accuracy and reduce the computing time. The proposed cascaded multi-layer utilizes a coarse-to-fine strategy to rapidly locate the tissues of interest and perform semantic segmentation to identify HSILs; at the coarse-level, fast localization of the tissues of interest is conducted, and at the fine-level, HSILs are identified based on the fast screening results of the tissues of interest. The framework of the proposed method is shown in Fig. 5.

### Cascaded multi-layer deep learning framework

For dealing with gigapixel data efficiently, each WSI is formatted into a tile-based pyramid data structure, which is denoted by $\mathcal {T}=\left\{ t_{l,i,j} \right\} _{~l=1,\ldots ,N}$ where *l* is the current level and *i*, *j* are the row and column index of a tile. $\mathcal {M}=\left\{ M_{1} ,\ldots ,M_{N}\right\}$ represents the set of deep-learning model $M_{l}$ at each layer, and an improved fully convolution network is developed as the deep learning-base model, which is described in the next section. The output of $M_{l}$ is the probabilities of HSILs $P_{l,i,j}$ at level *l*, which is used to produce an attention map $a_{l,i,j}$. A multi-layer attention map $\mathcal {A}=\{a_{l,i,j}\}_{~l=1,\ldots ,N-1}$ is computed to select tiles of interest $\mathcal {T'}=\left\{ t'_{l,i,j}\right\} _{~l=2,\ldots ,N}$ for further inspection at every next level $l+1$ by a tile selector model $\lambda$. The segmentation result of HSILs $\mathcal {S}_{N,i,j}(x,y)$ is generated by $M_{N}(t'_{N,i,j}(x,y))$.

For initialization, $t_{1,i,j}$ , i.e. the tiles in the first level, is processed by $M_{1}$ to generate the probabilities of HSILs $P_{1,i,j}$, as shown in Eq. (5).

5 $$\begin{aligned} P_{1,i,j}\left( x,y \right) =M_{1}\left( t_{1,i,j}\left( x,y \right) \right) \end{aligned}$$

The output of $M_{l}$ is the probabilities of HSILs $P_{l,i,j}$ at level *l*, which is used to produce an attention map $a_{l,i,j}$. If any pixel of $t_{l,i,j}$ has a probability greater than or equal to $\alpha$, set the attention map $a_{l,i,j}$ of that tile to 1, as shown in Eq. (6). In the practical case, $\alpha$ is set to 0.5.

6 $$\begin{aligned} a_{l,i,j}=\left\{ \begin{matrix} 1 &{}, max(P_{l,i,j}(x,y))\ge \alpha \\ 0 &{}, otherwise \end{matrix}\right. \end{aligned}$$

To render tiles of interest $t'_{l,i,j}$ in every next level, the attention map $a_{l-1}$ in the previous level $l-1$ is used by a tile selector model $\lambda _{l-1}$ with a mapping function as shown in Eq. (7). $a_{l-1,i,j}$, i.e. an attention map unit at $l-1$ level, is associated with $2^{2z}$ units at *l* level, and on the other hand, $a_{l,i,j}$ is associated with the attention map unit at $\left\lfloor i\times 2^{-z} \right\rfloor , ~\left\lfloor j\times 2^{-z} \right\rfloor$ at $l-1$ level. Thus, a tile $t_{l,i,j}$ is selected for further inspection as $t'_{l,i,j}$ when the corresponding attention map unit at the previous level $a_{l-1,~\left\lfloor i\times 2^{-z} \right\rfloor ,~\left\lfloor j\times 2^{-z} \right\rfloor }$ equals to 1.

7 $$\begin{aligned} t'_{l,i,j}=\lambda _{l-1}\left( a_{l-1,~\left\lfloor i\times 2^{-z}\right\rfloor ,~\left\lfloor j\times 2^{-z}\right\rfloor },t_{l,i,j} \right) =\left\{ \begin{matrix} t_{l,i,j} &{} ,a_{l-1,~\left\lfloor i\times 2^{-z} \right\rfloor ,~\left\lfloor j\times 2^{-z} \right\rfloor }=1\\ \phi , &{} otherwise \end{matrix}\right. \end{aligned}$$

In our implementation, each tile is associated with an attention tile, which contains 16 attention-units ($2^2 \times 2^2$ squares), and if the segmentation model at that level confirms any pixel of that tile associated to the attention unit(s) as the target, the attention units will be activated to select tiles for the next level.

For the subsequent levels $(l=\left[ 2, N \right] )$, $t'_{l,i,j}$ is processed by $M_{l}$ to generate the probabilities of HSILs $P_{l,i,j}$, as shown in Eq. (8). $P_{l,i,j}$ is then used to produce an attention map $a_{l,i,j}$ for identification of tiles of interest in the next level by a tile selector model $\lambda _{l}$ to generate $t'_{l+1,i,j}$ as formulated in Eqs. (6)–(7).

8 $$\begin{aligned} P_{l,i,j}\left( x,y \right) =M_{l}(t'_{l,i,j}(x,y)) \end{aligned}$$

In the level *N*, the selected tiles $t'_{N,i,j}$ produces probabilities $P_{N,i,j}$ by using (8). The segmentation result of HSILs $\mathcal {S}_{N,i,j}(x,y)$ is generated by $M_{N}$ using $t'_{N,i,j}(x,y)$ as shown in eq.(9).

9 $$\begin{aligned} \mathcal {S}_{N,i,j}\left( x,y \right) = \left\{ \begin{matrix} t'_{N,i,j}(x,y)&{}, ~M_{N}(t'_{N,i,j}(x,y))\ge \alpha \\ \phi &{}, otherwise \end{matrix}\right. \end{aligned}$$

### Modified FCN for segmentation of HSILs or higher (SQCC)

**Figure 6:**
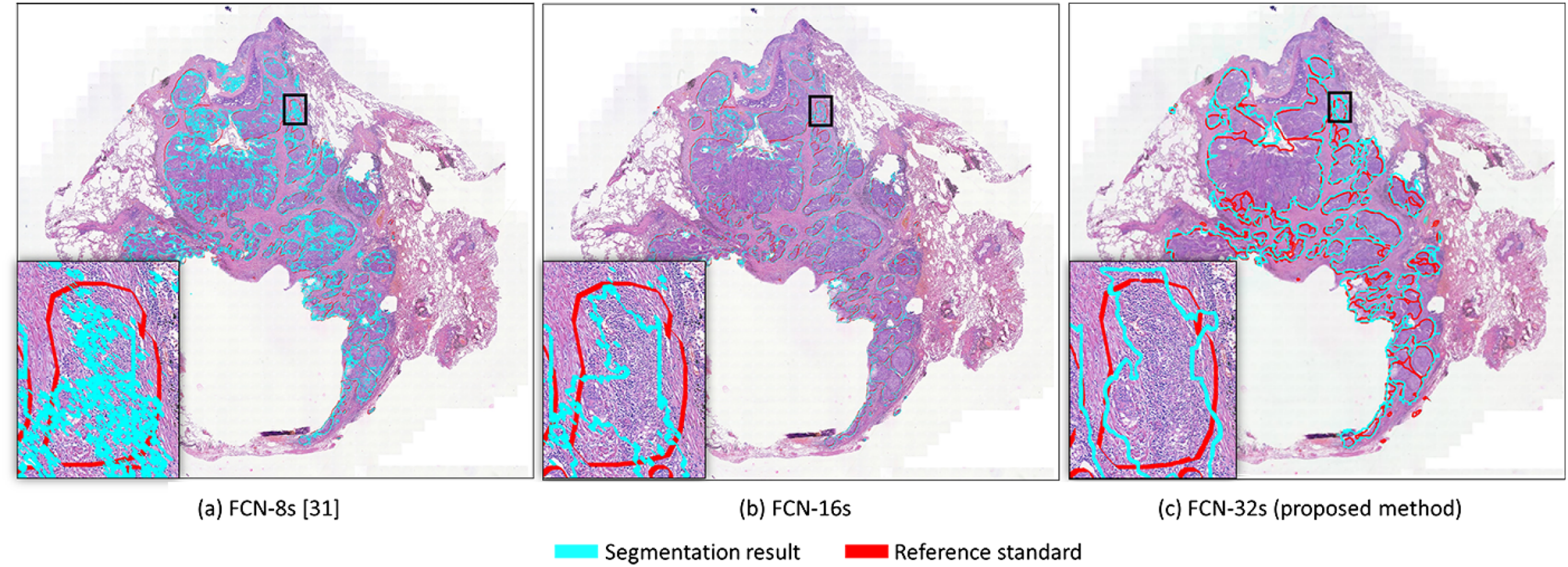
Comparison of segmentation results of three un-sampling layers of FCN. The results of (**a**) FCN-8s, and (**b**) FCN-16s are too fragmented. (**c**) In comparison, the results of FCN-32s are the most similar to reference standard.

Fully Convolutional Network (FCN) has been demonstrated to be effective in pathology, such as segmentation of nuclei in the images^47^, cell counting in different kinds of microscopy images^48^ and neuropathology^49^. The Fully Convolutional Network (FCN)^50^ is mainly composed of 18 convolution layers (each convolution layer is followed by a RELU layer), five pooling layers for downsampling, a SoftMax layer and three upsampling layers, namely FCN-8s, FCN-16s, and FCN-32s models forming a three-stream net where the outputs from each stream are aggregated to form the final output. The architecture of the proposed FCN as shown in Table 6. In our preliminary test using a lung dataset provided by Automatic Cancer Detection and Classification in Whole Slide Lung Histopathology challenge, which is held with the IEEE International Symposium on Biomedical Imaging (ISBI) in 2019^51^, we discover that single-stream FCN-32s could avoid overly fragmented segmentation results in comparison to the original three-stream net, as shown in Fig. 6. In addition, the cost of training and inference time is saved dramatically. In this study, we developed an improved FCN as the base deep learning model using the single-stream FCN-32s as the upsampling layers to improve segmentation result, lower GPU memory consumption, and speed-up the time for AI training and inference.

**Table 6:** The architecture of the proposed deep learning network.

Layer	Features (train)	Features (inference)	Kernel size	Stride
Input	512 $\times$ 512 $\times$ 3	512 $\times$ 512 $\times$ 3	–	–
Conv1_1	512 $\times$ 512 $\times$ 3	512 $\times$ 512 $\times$ 3	3 $\times$ 3	1 $\times$ 1
Conv1_2	710 $\times$ 710 $\times$ 64	710 $\times$ 710 $\times$ 64	3 $\times$ 3	1$\times$ 1
Pool1	710 $\times$ 710 $\times$ 64	710 $\times$ 710 $\times$ 64	2 $\times$ 2	2 $\times$ 2
Conv2_1	355 $\times$ 355 $\times$ 64	355 $\times$ 355 $\times$ 64	3 $\times$ 3	1 $\times$ 1
Conv2_2	355 $\times$ 355 $\times$ 128	355 $\times$ 355 $\times$ 128	3 $\times$ 3	1 $\times$ 1
Pool2	355 $\times$ 355 $\times$ 128	355 $\times$ 355 $\times$ 128	2 $\times$ 2	2 $\times$ 2
Conv3_1	178 $\times$ 178 $\times$ 128	178 $\times$ 178 $\times$ 128	3 $\times$ 3	1 $\times$ 1
Conv3_2	178 $\times$ 178 $\times$ 256	178 $\times$ 178 $\times$ 256	3 $\times$ 3	1 $\times$ 1
Conv3_3	178 $\times$ 178 $\times$ 256	178 $\times$ 178 $\times$ 256	3 $\times$ 3	1 $\times$ 1
Pool3	178 $\times$ 178 $\times$ 256	178 $\times$ 178 $\times$ 256	2 $\times$ 2	2 $\times$ 2
Conv4_1	89 $\times$ 89 $\times$ 256	89 $\times$ 89 $\times$ 256	3 $\times$ 3	1 $\times$ 1
Conv4_2	89 $\times$ 89 $\times$ 512	89 $\times$ 89 $\times$ 512	3 $\times$ 3	1 $\times$ 1
Conv4_3	89 $\times$ 89 $\times$ 512	89 $\times$ 89 $\times$ 512	3 $\times$ 3	1 $\times$ 1
Pool4	89 $\times$ 89 $\times$ 512	89 $\times$ 89 $\times$ 512	2 $\times$ 2	2 $\times$ 2
Conv5_1	45 $\times$ 45 $\times$ 512	45 $\times$ 45 $\times$ 512	3 $\times$ 3	1 $\times$ 1
Conv5_2	45 $\times$ 45 $\times$ 512	45 $\times$ 45 $\times$ 512	3 $\times$ 3	1 $\times$ 1
Conv5_3	45 $\times$ 45 $\times$ 512	45 $\times$ 45 $\times$ 512	3 $\times$ 3	1 $\times$ 1
Pool5	45 $\times$ 45 $\times$ 512	45 $\times$ 45 $\times$ 512	2 $\times$ 2	2 $\times$ 2
Drop6	23 $\times$ 23 $\times$ 512	16 $\times$ 16 $\times$ 512	–	–
Drop7	17 $\times$ 17 $\times$ 4096	10 $\times$ 10 $\times$ 4096	–	–
Upsampled	576 $\times$ 576 $\times$ 3	576 $\times$ 576 $\times$ 3	–	–
Output	512 $\times$ 512 $\times$ 3	512 $\times$ 512 $\times$ 3	–	–
